# Can Routine Blood and Urine Parameters Reveal Clues to Detect Bladder Cancer? A Case–Control Study

**DOI:** 10.3389/fonc.2021.796975

**Published:** 2022-01-21

**Authors:** Dan-Qi Wang, Juan Shuai, Hang Zheng, Zhong-Qiang Guo, Qiao Huang, Xiao-Feng Xu, Xiao-Dong Li, Hao Zi, Dao-Jing Ming, Xuan-Yi Ren, Xian-Tao Zeng

**Affiliations:** ^1^ Department of Urology, Zhongnan Hospital of Wuhan University, Wuhan, China; ^2^ Center for Evidence-Based and Translational Medicine, Zhongnan Hospital of Wuhan University, Wuhan, China; ^3^ Department of Geriatrics, Zhongnan Hospital of Wuhan University, Wuhan, China; ^4^ Department of Urology, Xianyang Central Hospital, Xianyang, China; ^5^ Department of Urology, Huaihe Hospital of Henan University, Kaifeng, China; ^6^ Institutes of Evidence-Based Medicine and Knowledge Translation, Henan University, Kaifeng, China; ^7^ Department of Urology, Kaifeng Central Hospital, Kaifeng, China

**Keywords:** bladder cancer, urinary biomarkers, diagnosis, blood test, urine test

## Abstract

**Objective:**

Limited attention has been paid to abnormal blood and urine test results for patients with bladder cancer. The present study aimed to identify whether blood and urine parameters are associated with bladder cancer.

**Methods:**

We used a case–control design and matched each patient with bladder cancer with three healthy controls of the same age and sex. Univariate conditional logistic regression was used to calculate the crude and adjusted odds ratio (OR) and its 95% CI. Multivariate conditional logistic regression was performed for confounders adjustment, and Spearman’s correlation coefficient was used to assess the correlation between tumor T stages and urine parameters.

**Results:**

Patients with bladder cancer (n = 360) and controls (n = 1050) were recruited. In the univariate conditional logistic analysis, higher urine pH was associated with a decreased risk of bladder cancer (OR = 0.67, 95% CI = 0.57–0.78), while higher values of urine protein (OR = 4.55, 95% CI = 3.36–6.15), urine glucose (OR = 1.56, 95% CI = 1.18–2.05), and urine occult blood (OR = 4.27, 95% CI = 3.44–5.29) were associated with an increased risk of bladder cancer. After adjustment for body mass index, fasting blood glucose, hypertension, red blood cells, white blood cells, lymphocytes, neutrophils, and platelets, significance still remained for urine pH (OR = 0.68, 95% CI = 0.53–0.88), urine protein (OR = 1.97, 95% CI = 1.21–3.19), urine glucose (OR = 2.61, 95% CI = 1.39–4.89), and urine occult blood (OR = 3.54, 95% CI = 2.73–4.58).

**Conclusion:**

This study indicated that lower urine pH and higher values of urine protein, urine glucose, and urine occult blood might be risk factors for bladder cancer.

## Introduction

Bladder cancer is the 11th most common cancer globally, with an estimated 550,000 new cases diagnosed in 2018 (1). The incidence rate, mortality rate, and disability-adjusted life-years (DALYs) rate of bladder cancer in men were 3.4 times, 3.3 times, and 3.3 times higher than those in women, respectively (2). Frequently, delayed and incomplete hematuria evaluation has resulted in the diagnosis being too late, especially for women, which has led to worse survival (3). By contrast, bladder cancer treatment is more expensive compared with the treatment of other cancers, and the cost increases with the higher severity of the disease at initial diagnosis (4). Therefore, early detection is crucial for the prognosis and cost-effectiveness of bladder cancer treatment.

Blood-based and urine-based diagnostic biomarkers have been developed to detect potential malignancies; however, none of them have been recommended for the diagnosis and follow-up of bladder cancer in clinical practice guidelines (5, 6). For example, although the use of a hematuria dipstick in high-risk populations has been reported, it was not recommended as a screening test because of the low incidence and short lead-time of bladder cancer (7). To improve poor outcomes, a previous case–control study identified multiple clinical features, such as visible hematuria, dysuria, and constipation to recognize early symptoms of bladder cancer (8). With consistently emerging technologies, we noticed that routine blood and urine parameters are often neglected when it comes to the recognition of cancer. Therefore, this study aimed to identify whether blood and urine parameters were associated with bladder cancer.

## Materials and Methods

### Study Design and Subjects

In this matched case–control study, we enrolled cases from the Bladder Cancer and Benign Prostatic Hyperplasia Study in Chinese Population (BPSC) database (9–11), and all patients provided written informed consent before enrollment. Only patients that were over 18 years old were eligible, and there was no restriction on tumor stage. Patients diagnosed with bladder cancer *in situ* were also included. The control group consisted of healthy people undergoing health examinations in the Zhongnan Hospital of Wuhan University. This research was reviewed and approved by the Committee for Ethical Affairs of the Zhongnan Hospital of Wuhan University.

### Data Collection

For the case group, data for analysis were obtained from patient records in the BPSC database, from which 431 bladder cancer patients were enrolled. To ensure matches, we randomly selected 4,310 available records from the health examination population. Observations would be excluded from further analysis if there was any missing value in their health information, such as sex; age; weight and height; fasting blood glucose (FBG; mmol/L); diagnosis of hypertension; counts of red blood cells (RBCs; 10^9^/L), white blood cells (WBCs; 10^9^/L), lymphocytes (10^9^/L), neutrophils (10^9^/L), and platelets (10^9^/L); urine pH; urinary protein (g/L); urinary glucose (mmol/L); urine occult blood; or tumor stage. Body mass index (BMI) was calculated as weight in kilograms divided by height in square meters (kg/m^2^). Thereafter, three controls within the same sex and 5-year age categories were selected and matched to each bladder cancer case.

### Statistical Analysis

Categorical variables, such as sex and hypertension, were expressed as counts and percentages, and continuous variables were expressed as the mean and SD. After descriptive statistics analysis, chi-squared tests and t-tests were adopted to identify confounding variables.

Univariate and multivariate conditional logistic regression analyses were conducted to measure the odds ratios (ORs) and 95% CIs, which examined associations between bladder cancer and urine pH, urinary protein, urinary glucose, and urine occult blood. A purposeful selection strategy of variables was used to build models. Based on biological rationales and reported risk factors of bladder cancer, potential confounding variables were considered and identified from previous comparative results. In addition, Spearman’s correlation coefficient was determined to assess correlations between tumor T stage and urine parameters.

A *p*-value of less than 0.05 was considered statistically significant, and all statistical analyses were accomplished using SAS 9.4 software (SAS, Cary, NC, USA).

## Results

**Figure 1:**
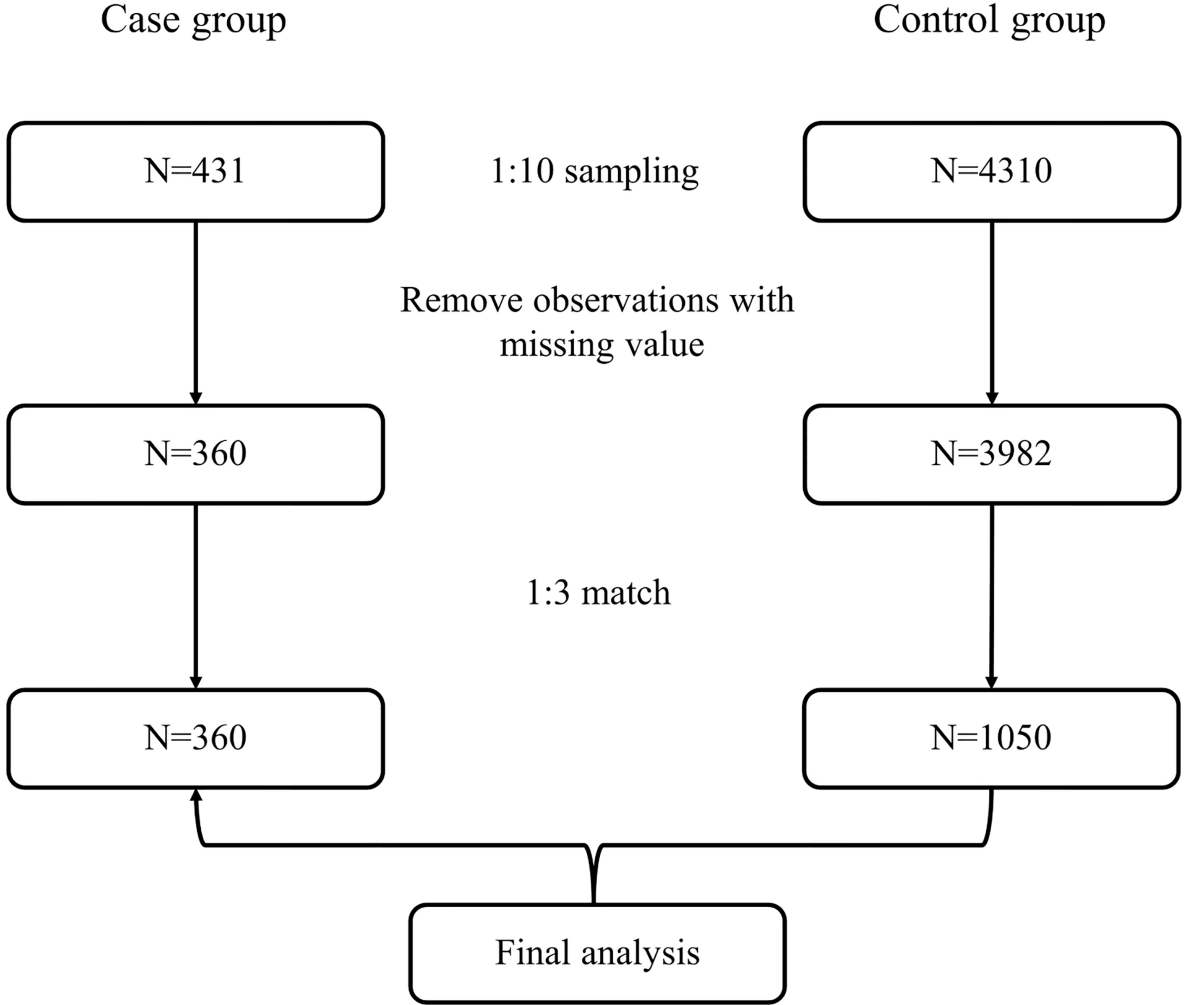
Flowchart of the selection of participants.

Ultimately, 360 cases and 1,050 matched controls were included (**Figure 1**). The demographic characteristics of the study participants are shown in **Table 1**. Statistical differences were observed for BMI and FBG (all *p*-values <0.001). The proportion of hypertension was different between cases and controls (36.39% *vs*. 47.90%, *p* < 0.001). With regard to routine blood parameters, only lymphocytes were reduced significantly in the case group compared with the control group (*p* < 0.001). The levels of serum total bilirubin, blood urea nitrogen, uric acid, and creatinine in the case group were higher compared with those in the control group (all *p*-values <0.001).

**Table 1:** Demographic characteristics and routine blood parameters of the study participants.

Characteristics	Case group(N = 360)	Control group(N = 1,050)	Chi-squared/t	*p*
Sex			–	Matched
Male	289 (80.28%)	841 (80.10%)		
Female	71 (19.72%)	209 (19.90%)		
Age (years)	65.38 ± 13.11	65.18 ± 13.18	–	Matched
BMI (kg/m^2^)	23.84 ± 3.24	24.60 ± 3.34	3.751	<0.001
FBG (mmol/L)	5.59 ± 1.29	5.94 ± 1.74	4.002	<0.001
Hypertension	131 (36.39%)	503 (47.90%)	14.367	<0.001
RBCs (10^9^/L)	4.41 ± 0.53	4.43 ± 0.44	0.665	0.507
WBCs (10^9^/L)	6.13 ± 1.79	6.32 ± 1.64	1.750	0.081
Lymphocytes (10^9^/L)	1.61 ± 0.56	2.11 ± 0.62	14.188	<0.001
Neutrophils (10^9^/L)	4.03 ± 3.09	3.78 ± 1.37	−1.514	0.131
Platelets (10^9^/L)	193.57 ± 57.73	191.80 ± 52.45	−0.514	0.608
Serum total bilirubin (μmol/L)	14.32 ± 6.56	12.65 ± 6.24	−4.323	<0.001
Blood urea nitrogen (mmol/L)	6.06 ± 2.20	5.19 ± 1.60	−6.861	<0.001
Uric acid (μmol/L)	352.43 ± 100.08	304.58 ± 79.05	−8.233	<0.001
Creatinine (μmol/L)	86.64 ± 32.03	77.70 ± 20.17	−4.967	<0.001

BMI, body mass index; FBG, fasting blood glucose; RBCs, red blood cells; WBCs, white blood cells.

**Table 2** shows the results of univariate conditional logistic regression for urine parameters. The level of urine pH revealed a strong inverse relationship with the risk of bladder cancer (OR = 0.67, 95% CI = 0.57–0.78, *p* < 0.001). By contrast, higher levels of urine protein (OR = 4.55, 95% CI = 3.36–6.15, *p* < 0.001), urine glucose (OR = 1.56, 95% CI = 1.18–2.05, *p* = 0.002), and urine occult blood (OR = 4.27, 95% CI = 3.44–5.29, *p* < 0.001) were associated with an increased risk of bladder cancer.

**Table 2:** Univariate conditional logistic regression for urine parameters.

Exposures	Case group (N = 360)	Control group (N = 1,050)	OR (95% CI)^*^	*p*
Urine pH	5.92 ± 0.64	6.17 ± 0.84	0.67 (0.57, 0.78)	<0.001
Urine protein (g/L)	0.47 ± 0.71	0.09 ± 0.36	4.55 (3.36, 6.15)	<0.001
Urine glucose (mmol/L)	0.13 ± 0.61	0.05 ± 0.30	1.56 (1.18, 2.05)	0.002
Urine occult blood	1.30 ± 1.22	0.18 ± 0.50	4.27 (3.44, 5.29)	<0.001

^*^ OR, odds ratio.

**Table 3** shows the results of multivariate conditional logistic regression analysis with confounders adjustment. After adjustment for BMI, FBG, hypertension, RBCs, WBCs, lymphocytes, neutrophils, and platelets in model 2, significant differences remained for urine pH (OR = 0.68, 95% CI = 0.53–0.88), urine protein (OR = 1.97, 95% CI = 1.21–3.19), urine glucose (OR = 2.61, 95% CI = 1.39–4.89), and urine occult blood (OR = 3.54, 95% CI = 2.73–4.58). In model 3, the levels of urine protein, urine glucose, and urine occult blood were associated positively with bladder cancer when further adjusted by total bilirubin, blood urea nitrogen, uric acid, and creatinine (all *p*-values <0.05).

**Table 3:** Multivariate conditional logistic regression analysis with confounders adjustment.

Exposures	Model 1		Model 2		Model 3	
	OR (95% CI)	*p*	OR (95% CI)	*p*	OR (95% CI)	*p*
Urine pH	0.66 (0.53, 0.83)	<0.001	0.68 (0.53, 0.88)	0.003	0.79 (0.60, 1.04)	0.092
Urine protein (g/L)	2.11 (1.39, 3.19)	<0.001	1.97 (1.21, 3.19)	0.006	1.83 (1.06, 3.16)	0.031
Urine glucose(mmol/L)	2.43 (1.46, 4.07)	<0.001	2.61 (1.39, 4.89)	0.003	2.86 (1.31, 6.26)	0.008
Urine occult blood	3.47 (2.75, 4.38)	<0.001	3.54 (2.73, 4.58)	<0.001	3.67 (2.78, 4.86)	<0.001

Model 1: Adjusted for BMI, FBG, and hypertension. Model 2: Further adjusted for RBCs, WBCs, lymphocytes, neutrophils, and platelets based on model 1. Model 3: Further adjusted for serum total bilirubin, blood urea nitrogen, uric acid, and creatinine based on model 2.

BMI, body mass index; FBG, fasting blood glucose; RBCs, red blood cells; WBCs, white blood cells.

Among the 360 cases included, 62 were at T0/Ta/Tis stage (17.22%), 201 were at the T1 stage (55.83%), 64 were at T2/T2a/T2b stage (17.78%), 3 were at T3/T3a/T3b stage (0.83%), and 30 had missing T stage data (8.33%). The results presented in **Table 4** show that the T stage was associated positively with urine protein and urine occult blood levels (both *p*-values <0.05).

**Table 4:** Spearman’s correlation coefficients between T stage and urine parameters.

Variable	Urine pH (*p*)	Urine protein (*p*)	Urine glucose (*p*)	Urine occult blood (*p*)
T stage	0.06 (0.26)	0.17 (<0.01)	−0.03 (0.60)	0.16 (<0.01)

## Discussion

In the sample population, 80% of the patients were male, and the sex ratio conformed to the results of an epidemiological investigation on bladder cancer cases, which collected data from 501 cancer registries in China (12). In the present study, the average BMI of the control group was 24.60 kg/m^2^, which was beyond the upper normal limit (23.9 kg/m^2^). Statistical significance was observed for the comparison of the BMI between cases and controls (*p* < 0.001). By contrast, three Swedish cohorts, which included 340,000 men, indicated that BMI (hazard ratio (HR) = 1.02, 95% CI = 0.98–1.08) and systolic blood pressure (HR = 0.98, 95% CI = 0.94–1.02) showed no statistically significant associations with bladder cancer outcomes (13). A prospective pooled cohort study stated that the effects of BMI on bladder cancer varied between males and females. BMI showed a positive association with risk for non-muscle invasive bladder cancer among men (HR = 1.09, 95% CI = 1.01–1.18) but was inversely associated with bladder cancer risk among women (HR = 0.90, 95% CI = 0.82–0.99) (14). Based on the evidence described above, Teleka et al. indicated that estimating bladder cancer risk using BMI values might depend considerably on the composition of the study population in terms of sex and tumor T stage (14). Males made up 80% of our sample population, and 73.05% of cases were staged as T0, Ta, Tis, or T1, which could partly explain the divergence between our results and other studies.

Previous studies have reported a positive association between bladder cancer risk and a higher level of FBG (15), and it was not uncommon that patients with diabetes had a higher risk of bladder cancer (16). Nevertheless, in the present study, the FBG of the control group was significantly higher than that of the bladder cancer cases (*p* < 0.001), and the average values of both groups were no more than 6 mmol/L. To support the survival and proliferation of cancer cells, increased glucose uptake is achieved through glucose transporter type 1 (GLUT-1) translocation to the membrane (17), which might account for the decrease in the FBG level in bladder cancer cases. Inhibitors of the sodium-glucose cotransporter-2 (SGLT-2) are increasingly used to treat type 2 diabetes and were reported to be positively associated with the risk of bladder cancer (OR = 3.97, 95% CI = 3.39–4.66) (18). A study of the metabolic effects of bladder cancer in 22 patients and 10 controls reported a lower glucose clearance accompanied by higher insulin levels, which indicated a state of insulin resistance (19). However, that study was limited by the sample size and the completely different sex balance of the cases and controls. The complex interactions of blood glucose and bladder cancer at different stages remain unclear and require further study.

The complicated interactions between tumors and inflammatory responses (20) have led to hematological inflammation-associated phenomena being used widely to predict the biological behavior of various tumors, including urothelial carcinoma. With regard to bladder cancer, studies have demonstrated the prognostic value of blood cell count, especially the neutrophil-to-lymphocyte ratio (NLR) and the platelet-to-lymphocyte ratio (PLR). For example, it was reported that a higher NLR was associated with an increased risk of disease recurrence and progression in patients with bladder cancer (21, 22). Zhao et al. indicated that tumor tissue stimulated the production of neutrophils, which could promote tumor cell proliferation and angiogenesis by releasing cytokines and effector molecules, especially interleukins and the pro-angiogenic vascular endothelial growth factor (23). Meanwhile, a decreased lymphocyte level could weaken adaptive immune responses towards the tumor. The combined effects of these two cellular mechanisms indicated that the NLR reflected the results of antagonism between inflammatory responses and antitumor effects and therefore had a certain prognostic value for tumors (23). Blood cell count is routinely tested for hospitalized patients, so it required no additional cost or patient inconvenience (22). Such test results could help clinicians to make targeted treatment plans and follow-up schedules as supplements to imaging and cystoscopy (24). Notably, the antitumor and protumor phenotypes of tumor-associated neutrophils (TANs) are regulated by microenvironmental factors (25–27); therefore, the phenotypes and effects of TANs could vary in different histological types of bladder cancer (28).

According to the results shown in **Table 3**, the OR values were relatively stable in our three models after adjustment. Our results indicated a consistently positive correlation between lower urine pH and the risk of bladder cancer, except in model 3, in which uric acid was adjusted. According to previous studies, the independent effects of urine pH on bladder cancer risk are unclear; however, it was demonstrated that urine pH could intensify the impact of tobacco exposure (29, 30). Alguacil et al. found that acidic urine (all tested urine pH values ≤6.0) was not associated with the risk of bladder cancer among non-smokers (OR = 1.0, 95% CI = 0.6–1.8) but was strongly associated among current smokers (OR = 2.1, 95% CI = 1.3–3.2) (30). Moreover, a population with comparable cigarette smoking intensity had an increased risk of bladder cancer with acidic urine, and an interaction existed between consistent acidic urine and heavy smoking (*p*_interaction_ = 0.024) (30). High levels of tobacco exposure generate *N*-glucuronidated arylamine carcinogens, which are synthesized in the liver and excreted *via* the kidneys and bladder. As the medium in which the bladder mucosa is exposed to carcinogens, urine is important in the development of bladder cancer, especially when it carries carcinogens (31). *N*-Glucuronide conjugates of aromatic amines are relatively inactive at neutral pH but would be rapidly hydrolyzed to active free aromatic amines and covalently bind to urothelial DNA under acidic conditions (30, 32). For instance, 50% of the *N*-glucuronides of benzidine were hydrolyzed after 5 min at pH 5.3, 25 min at pH 6.3, and 140 min at pH 7.4 (33). Rothman et al. studied urine samples from Indian workers who were exposed to benzidine occupationally (34), and urine pH was found to have an inverse correlation with the proportion of free benzidine and associated metabolites (*p* < 0.001). In exfoliated urothelial cells, workers with urine pH < 6.0 had 10-fold higher levels of DNA adducts than those with urine pH ≥ 7.0 (*p* = 0.0037) (34). In summary, acidic urine could facilitate the process of carcinogen activation and tumorigenesis. For a population exposed to aromatic amines occupationally or through smoking, low urine pH could be a particularly important risk factor for bladder cancer (29).

As shown in **Table 3**, urine occult blood was strongly associated with the risk of bladder cancer, with OR values ranging from 3.47 to 3.67 in models 1, 2, and 3 (all *p*-values <0.001). Meanwhile, a significant correlation was observed between T stages and urine parameters (*p* < 0.01) (**Table 4**), which indicated invasive tumor biological behavior. As the most common symptom of bladder cancer, sometimes the only one, hematuria could be a sign of underlying urological malignancy. It is strongly recommended for patients with symptoms suggestive of bladder cancer to undergo a cystoscopy, which cannot be replaced by cytology or any other non-invasive test in the diagnosis of bladder cancer (7). However, according to a retrospective study, only 25 (1.2%) among 2,118 patients were diagnosed with bladder cancer using cystoscopy for asymptomatic microscopic hematuria (35). Given that cystoscopy is an invasive and expensive test procedure, its poor detection rate has limited the application of hematuria as a screening test for bladder cancer (35). Two directions were explored to resolve this contradiction. One was to develop non-invasive screening tests in high-risk populations (36) that meet the ASSURED criteria (Affordable, Sensitive, Specific, User-friendly, Rapid, Equipment free, and Deliverable) framed by the WHO. The other was to identify more clinical features associated with bladder cancer, which were used to build accurate prediction models for the diagnosis and prognosis of bladder cancer. Both directions tried to avoid suffering and costly evaluations in relatively low-risk populations (37).

The present study had some limitations. The practical benefit of each independent parameter was confined. However, we failed to build predictive models because of limited data. Based on multiple clinical features, further studies could use a nomogram to predict the occurrence of bladder cancer cases with high accuracy. Besides, we did not conduct subgroup analysis; therefore, the effects of smoking and sex remain unknown.

## Conclusion

In this study, we identified and interpreted significant associations between bladder cancer and certain blood and urine parameters. Urine pH, urine protein, urine glucose, and urine occult blood were found to be associated with the risk of bladder cancer and remained significant after adjusting for BMI, FBG, hypertension, RBCs, WBCs, lymphocytes, neutrophils, and platelets. These parameters might be useful in the early detection of bladder cancer. While NLR and urine occult blood have been widely discussed, urine pH, urine protein, and urine glucose are undervalued. This study provides new insight into routine blood and urine parameters and demonstrated their value in the diagnosis and follow-up of bladder cancer.

## Data Availability Statement

The raw data supporting the conclusions of this article will be made available by the authors, without undue reservation.

## Ethics Statement

The studies involving human participants were reviewed and approved by the Committee for Ethical Affairs of the Zhongnan Hospital of Wuhan University. The patients/participants provided their written informed consent to participate in this study.

## Author Contributions

D-QW, X-FX, and X-TZ designed this study. JS, Z-QG, HZh, and D-JM collected the data. X-DL and HZi re-checked the data. HZi and QH performed the analysis. D-QW and Z-QG wrote the manuscript. X-YR and X-TZ reviewed the manuscript.

## Funding

This work was supported by the National Key Research and Development Plan of China (Technology helps Economy 2020; 2016YFC0106300), without any financial interest or benefit.

## Conflict of Interest

The authors declare that the research was conducted in the absence of any commercial or financial relationships that could be construed as a potential conflict of interest.

## Publisher’s Note

All claims expressed in this article are solely those of the authors and do not necessarily represent those of their affiliated organizations, or those of the publisher, the editors and the reviewers. Any product that may be evaluated in this article, or claim that may be made by its manufacturer, is not guaranteed or endorsed by the publisher.
